# Withdrawal Symptoms Following Discontinuation of Vortioxetine—Retrospective Chart Review

**DOI:** 10.3390/ph14050451

**Published:** 2021-05-11

**Authors:** Marcin Siwek, Adrian Andrzej Chrobak, Aleksandra Gorostowicz, Anna Julia Krupa, Dominika Dudek

**Affiliations:** 1 Department of Affective Disorders, Jagiellonian University Medical College, Kopernika Street 21a, 31-501 Kraków, Poland; 2 Department of Adult Psychiatry, Jagiellonian University Medical College, Kopernika Street 21a, 31-501 Kraków, Poland; adrian.chrobak@uj.edu.pl (A.A.C.); dominika.dudek@uj.edu.pl (D.D.); 3 Department of Psychiatry, Jagiellonian University Medical College, Kopernika Street 21a, 31-501 Kraków, Poland; aleksandra.gorostowicz@doctoral.uj.edu.pl (A.G.); annajuliakrupa@doctoral.uj.edu.pl (A.J.K.)

**Keywords:** discontinuation symptoms, vortioxetine, antidepressants, withdrawal, retrospective chart review

## Abstract

The efficacy of vortioxetine has been proven in many studies, but data concerning discontinuation symptoms (DS) after vortioxetine withdrawal is scarce. The aim of our study is to systematically evaluate the prevalence, determinants, and clinical features of vortioxetine DS in a retrospective chart review. Data were obtained from medical records of 263 adult patients with depressive disorders who discontinued former vortioxetine treatment. DS were observed in eight (3%) patients after 71–375 days (median 272) of treatment. DS emerged after median three days following vortioxetine withdrawal and lasted for median seven days. The clinical presentation of DS involved: emotional lability (100% of patients), irritability (75%), sudden worsening of mood (75%), nervousness (37.5%), and agitation (37.5%). Median DESS score was four (range of four to six). DS were significantly more prevalent after accidental vs. planned discontinuation (adjusted *p* = 0.011) and were less frequent after switching to a different antidepressant vs. ceasing pharmacotherapy (adjusted *p* = 0.0165). DS appeared more often if patients discontinued therapy without medical consultation (adjusted *p* = 0.033). The occurrence of DS was not associated with the dose and way of drug discontinuation (sudden vs. gradual). In sum, our results show that clinicians should be aware that vortioxetine withdrawal is associated with the possibility of DS.

## 1. Introduction

Antidepressants are among the most commonly used psychiatric medications [1]. Duration of antidepressant administration depends on the diagnosis, patient’s condition, and treatment tolerance. The emergence of symptoms upon treatment interruption is called discontinuation/withdrawal syndrome. It was first reported in association with imipramine in 1959 [2]. Withdrawal syndrome usually occurs in patients taking antidepressants for longer than six to eight weeks [3,4], and its risk is increased by longer duration of treatment and higher doses administered [5,6,7,8]. Discontinuation symptoms (DS) may appear as soon as on the first day after stopping the antidepressant or reducing the daily dose (usually within three to four days [9]; onset of symptoms after one week is uncommon [7]). Gradual dose reduction, called tapering, limits but does not eliminate the risk of developing DS completely [9,10]. DS are usually mild to moderate [5,6,7] and resolve spontaneously after five days to three weeks [4,6] but in some cases may persist for months or even years [9,10]. In severe cases, reintroducing the antidepressant or starting a new one may be of help [4,5]. It was reported that cognitive behavioural therapy is efficacious in reducing patients’ distress due to DS.

The risk of DS upon drug withdrawal is linked to the vast majority of antidepressants. The list of possible DS presentations is long and includes both psychiatric and somatic symptoms [3,4,5,7]. For selective serotonin reuptake inhibitors (SSRIs) and serotonin-norepinephrine reuptake inhibitors (SNRIs—venlafaxine, duloxetine), the most commonly reported DS are flu-like symptoms (shivering, muscle pain, fatigue, excessive sweating), headaches, weakness, vertigo, gait imbalance, dizziness, ataxia, tremors, paresthesia, nausea, vomiting, diarrhea, abdominal pain, electric-shock-like experiences in the brain (referred to as brain zaps), visual disturbances, insomnia, vivid dreams, nightmares, agitation, irritability, anxiety, tearfulness, and sexual dysfunctions [7,10,11,12]. In the case of tricyclic antidepressants withdrawal, sensory abnormalities and problems with equilibrium appear to be less common while more frequently reported DS include headaches, gastrointestinal effects, affective symptoms, sleep disturbances, and flu-like symptoms [4,6,7]. When halting the treatment with the classical, irreversible monoamine oxidase inhibitors, reported DS are more severe and may include hallucinations, delusions, delirium, worsening of depressive symptoms, anxiety, agitation, and insomnia [7,13]. Reactions to moclobemide discontinuation appear to be very rare—a single case of a patient with flu-like symptoms lasting seven days after sudden reduction of a high dose of moclobemide has been reported [13]. For mirtazapine, the manufacturer warns of adverse reactions upon the drug discontinuation [14]. A few cases of DS after mirtazapine discontinuation have been reported [15,16]. Mianserine appears to be relatively safe—a single case of panic anxiety after abrupt cessation of mianserine has been documented [17]. Until now, there are no clinical studies confirming DS after agomelatine withdrawal [18]. A wide variety of rare DS after antidepressants have been reported, e.g., extrapyramidal symptoms [19], manic/hipomanic episode [15,20], delirium [5,7,21,22], and cataplexy [23]. What is more, cases of severe DS (resembling stroke) after venlafaxine and paroxetine are documented [24,25].

Yet, to the best of our knowledge, there are no reports or clinical cases of patients with discontinuation symptoms after stopping vortioxetine, a new antidepressant (approved for the treatment in 2013) with multimodal activity. However, the occurrence of withdrawal symptoms was evaluated in clinical studies. Jacobsen et al. [26] and Baldwin et al. [27] conducted trials indicating that the risk after two weeks of vortioxetine withdrawal was comparable to that observed after placebo cessation. The aim of our study is to systematically evaluate the prevalence and clinical features of vortioxetine DS in a retrospective chart review.

## 2. Results

The drug was discontinued in 263 patients. The clinical description of the studied groups is presented in Table 1. Withdrawal symptoms were observed in the subgroup of eight patients (3%). The median age of participants who reported DS was 31 (27–52.8). They were five females and three males. Vortioxetine was used in monotherapy in five of these patients (62.5%). Three patients (37.5%) were under polypharmacotherapy: the first patient was receiving lamotrigine 200 mg/daily; the second, opipramol 50 mg/daily, and the third, bupropion 150 mg/daily. In those patients, vortioxetine was the only drug that was discontinued. Six patients were treated with 10 mg and two with 15 mg of vortioxetine (mean dose 11.25 mg (SD = 2.32)). The median duration of pharmacotherapy prior to drug discontinuation was 272 days (71–375). Five patients (62.5%) discontinued treatment gradually and in agreement with their physician. Three individuals (37.5%) discontinued suddenly and without acquiring the medical advice beforehand. The median time between vortioxetine withdrawal and the emergence of DS was 3 days (1.25–4.75). Every patient scored at least 4 points in the DESS inventory. The median DESS score was four (range of four to six) [28]. In the case of five patients, DS resolved spontaneously after 3–10 days (median 7 days, (5–8.5)). In the group of three individuals, symptoms subsided within one to two days after reintroduction of vortioxetine. In Table 2, we have summarized the most common DS. The clinical presentation of discontinuation syndrome was similar—the following symptoms were most frequently reported: lability (100%), irritability (75%), sudden worsening of mood (75%), nervousness (37.5%), and agitation (37.5%). All of the patients presenting DS reported that the symptoms caused significant discomfort and impaired their functioning.

The group of individuals without withdrawal syndrome consisted of 255 patients. Their median age was 39 (30–51). They were 143 females and 112 males. Vortioxetine was used in monotherapy in 60 (23.5%) patients. In the group of 195 patients (76.5%) vortioxetine was used in polytherapy. In those cases, vortioxetine was the only drug that was discontinued. A total of 135 patients (58.4%) stopped the medication due to lack of effectiveness, 40 patients (17.3%) due to side effects, 32 patients (13.9%) because of symptomatic improvement or symptoms remission, 2 patients (0.9%) discontinued vortioxetine accidentally, and 22 (9.5%) due to other reasons. Mean vortioxetine dose was 10.58 (SD = 2.38). The median duration of vortioxetine treatment prior to drug discontinuation was 74 days (38.8–170.3). The discontinuation method was gradual in the case of 208 patients (81.6%) and was performed after obtaining medical advice in 47 individuals (18.4%).

There was a significant association between the way of deciding on discontinuation of treatment (with/without medical consultation) and occurrence of withdrawal symptoms (*p* = 0.009, df = 1). The odds of occurrence of withdrawal symptoms were 7.376 (95% CI: 1.703–31.949) times higher if vortioxetine was discontinued without prior medical consultation than after consulting the physician. An association between the duration of pharmacotherapy prior to discontinuation and the occurrence of discontinuation symptoms was observed. Patients with withdrawal symptoms had significantly longer duration of treatment (median 272 days (71–375)) than patients without the symptoms (74 days (38.75–170.25)) (*p*_MW_ = 0.032, U = 1469.5, z = 2.149, estimated effect size: r = 0.133, *n* = 262 [29]). The analysis showed statistically significant associations between the occurrence of withdrawal symptoms and the reason for discontinuation of the drug. In the group of patients with DS, vortioxetine was more often discontinued accidentally and less commonly due to lack of effectiveness (FFH = 29.198, Cramer’s V = 0.619, df = 4, *p*_FFH_ < 0.001, *n* = 238). There was a significant relationship between the type of pharmacotherapy (monotherapy/polytherapy) and the occurrence of withdrawal symptoms (*p*_Fisher’sexacttest_ = 0.024, df = 1). Odds ratio: 5.417 (95%CI: 1.258–23.331)—odds of occurrence of withdrawal symptoms were 5.417 times higher if vortioxetine was used in monotherapy. There was a significant association between the occurrence of withdrawal symptoms and the medication switch category during discontinuation of vortioxetine. Withdrawal symptoms appeared less frequently when vortioxetine was switched to another antidepressant (FFH (df = 2) = 10.558, Cramer’s V = 0.213, *p*_FFH_ = 0.003, *n* = 247). After implementing false discovery rate (FDR) *p*-value correction with Benjamin–Hochberg procedure for multiple comparisons, three of the above-mentioned parameters remained statistically significantly associated with the occurrence of withdrawal symptoms: (1) reason for discontinuing of vortioxetine (*p* < 0.001, adjusted *p* = 0.011)—vortioxetine was more often discontinued accidentally and less commonly due to lack of effectiveness, (2) switch to different antidepressant medication or halting antidepressant pharmacotherapy (*p* = 0.003, adjusted *p* = 0.0165), and (3) the way of deciding on discontinuation of treatment (with/without medical consultation) (*p* = 0.009, adjusted *p* = 0.033).

We have shown that occurrence of withdrawal symptoms was not significantly associated with patients age (U = 812, p_Mann–Whitney_ = 0.326, z = −0.982 estimated effect = −0.06), sex (*p*_Fisher’sExactTest_ > 0.999 df = 1, OR female vs. male: 1.305 (95% CI: 0.035–5.579)), rate of vortioxetine discontinuation (sudden or gradual, p_Fisher’sExactTest_ = 0.179, df = 1, OR sudden vs gradual: 0.377 (95% CI: 0.087–1.631)), dose (*p*_Mann–Whitney_ = 0.057, U = 1144, estimated effect size = 0.12, *n* = 251), somatic comorbidities (*p*_Fisher’sExactTest_ = 0.212, df = 1, OR: 2.400 (95% CI: 0.555–10.374)), number of psychiatric comorbidities (p_FFHTest_ = 0.947, FFH = 1.141, df = 4, Cramer’s V = 0.066 (*p* = 0.916)), and presence of comorbid anxiety disorders (p_Fisher’sExactTest_ = 0.725, df = 1, OR:1.383 (95%CI: 0.338–5.654)).

## 3. Discussion

In this study, we have evaluated the prevalence and clinical characteristics of vortioxetine DS and their determinants in a group of 445 patients in a naturalistic setting. Analyses indicate that DS are relatively rare, occurring in 8 (3%) out of 263 patients who discontinued treatment. In the group of patients with DS, vortioxetine was more commonly discontinued accidentally and without prior medical consultation. The withdrawal symptoms appeared less frequently when vortioxetine was switched to another antidepressant or in cases when the drug was not effective. The occurrence of vortioxetine DS was not associated with patients’ age, sex, method of vortioxetine discontinuation (sudden or gradual), dose, as well as the presence of somatic and psychiatric comorbidities, including comorbid anxiety disorders. After implementing adjustments for multiple comparisons, we also showed that the occurrence of DS was not associated with polytherapy and duration of treatment with the use of vortioxetine. The clinical presentation of discontinuation syndrome was similar—the following symptoms were reported: irritability, lability, tearfulness, and feeling of inner tension.

The Food and Drug Administration (FDA) published a review of vortioxetine on its website, where it warns of potential DS when abruptly stopping vortioxetine after taking it in a daily dose of 15 mg or 20 mg [30]. In placebo-controlled trials, patients experienced DS such as headache, muscle tension, mood swings, outbursts of anger, dizziness, and running nose after cessation of vortioxetine in the above-mentioned doses [30]. In our study, six out of eight patients with vortioxetine DS symptoms received a daily dose of 10 mg, while the two remaining patients received 15 mg and 20 mg of vortioxetine a day. Moreover, our analysis showed no significant associations between drug dose and frequency of DS, indicating that withdrawal symptoms may also affect patients treated with doses lower than 15 mg a day. Noteworthy, two patients developed DS despite the gradual withdrawal of vortioxetine and taking a reduced dose (5 mg) for 7 to 14 days before discontinuing treatment. In the case of five patients, DS resolved spontaneously (after different duration: from 3 days to 10 days) and in three patients, after reintroduction of initial vortioxetine dosage within one to two days. On the other hand, Jacobsen et al. [26] and Baldwin et al. [27] conducted trials indicating that the risk after two weeks of vortioxetine withdrawal was comparable to that observed after placebo cessation. Placebo-comparable expression of withdrawal symptoms mentioned in previous studies is most likely due to a short follow-up period. It should be noted that in our study, patients in whom DS was observed were treated for a significantly longer time (median 272 days). The scarcity of data signifies the need for further research that would take into account the whole range of recommended vortioxetine doses, long follow-up period, and the need to control the placebo effect.

We presented the clinical characteristics of eight cases with vortioxetine DS. The symptomatology of discontinuation syndrome was similar—patients most often reported: lability (100% patients), irritability (75%), sudden worsening of mood (75%), nervousness (37.5%), and agitation (37.5%). These complaints are to a degree similar to the affective symptoms of withdrawal syndrome associated with the use of antidepressants from other groups: SSRIs, SNRIs, and tricyclic drugs. Subjects in our study also reported somatic symptoms, such as pressure in the stomach, physical weakness, and headache, although these were rare. Patients’ DESS scores ranged between four and seven points, which reflects a relatively low variety of symptoms. However, it should be noted that this scale was developed for withdrawal from SRRI antidepressants. Hence, the use of this inventory in order to evaluate the newly described syndrome associated with withdrawal of vortioxetine (serotonin modulator and stimulator characterised by different, more complex mechanism of action compared to SSRIs) may, in our opinion, be inaccurate, especially given the fact that patients rarely presented somatic symptoms, which account for a significant proportion of the DESS score and reported symptoms not included in this tool (anergy, physical weakness, apathy, and amotivation). Moreover, this tool in itself has some limitations because it does not cover all of the characteristic DS, and the measured symptoms are non-specific [28].

The research on the neurobiological pathophysiology of DS is still sparse. Amongst the suggested mechanisms are the dysregulation of preexisting balance between neuromediators in the brain (serotonin, norepinephrine, dopamine, acetylcholine, and gamma-aminobutyric acid GABA), changes in hippocampal *N*-methyl-D-aspartate (NMDA) receptor density, and certain genetic vulnerabilities [10]. DS are more likely to occur with antidepressants with a shorter half-life and no active metabolites [5]. Thus, amongst SSRIs, the risk of DS is highest after stopping paroxetine (half-life of around 24 h, no active metabolites, anticholinergic activity) and relatively low when discontinuing fluoxetine (antidepressant metabolised to norfluoxetine with half-life up to 16 days) [4,31]. Venlafaxine, a medication with a short half-life (around 5 h) with influence on both serotonergic and adrenergic transmission, has the potential to generate DS even after skipping one dose of the drug [24].

Vortioxetine is an inhibitor of serotonin transporter, an agonist of 5HT1A receptor, a partial agonist of 5HT1B receptor, and an antagonist of 5HT1D, 5HT3, and 5HT7 receptors. It is metabolised by cytochrome P450 2D6 isoenzyme to inactive metabolites. The drug’s half-life is 57–66 h [32]. The relatively long half-life is a characteristic that theoretically should minimize the risk of DS appearance, whereas not having active metabolites is considered to magnify it [4,5]. Vortioxetine’s maximum plasma concentration (C_max_) is observed 7–11 h after administration (T_max_). The absolute bioavailability for vortioxetine is high, up to 75% (both after intravenous and oral administration) [32]. Inhibition of serotonin re-uptake is a common mechanism of action for both vortioxetine and other antidepressants that might cause clinically similar DS upon cessation.

Knowledge of antidepressant DS is extremely important because of their potential for misdiagnosis leading to incorrect therapeutic decisions. It is important to notice that DS may be misdiagnosed as adverse effects of the new medication if they follow an antidepressant switch. However, our results indicate that withdrawal symptoms upon vortioxetine treatment cessation were significantly less common during a switch to different antidepressant medication. Discontinuation reactions may be incorrectly considered to be a recurrence of the basic underlying psychiatric illness. A patient’s non-compliance to antidepressant treatment sometimes results in the development of DS, which can be interpreted by the doctor as worsening of the patient’s condition and lead to the conclusion that the treatment is ineffective. In our study, DS were most commonly present in subjects who stopped the medication by accident or made the decision to discontinue vortioxetine without consulting their psychiatrist first. As mentioned in the introduction, manic/hipomanic episodes can occur as an antidepressant DS. Their emergence may incorrectly change the patient’s diagnosis from unipolar depression to bipolar I or II disorder. Finally, DS may resemble many somatic disorders, giving rise to unnecessary diagnostic interventions [7].

We are aware of the several limitations of our study: (a) the rare occurrence of vortioxetine DS resulted in a relatively small number of subjects in that group, hindering statistical evaluation of the features related to DS emergence; (b) the heterogeneity of the studied group; (c) the variability of vortioxetine dosage among studied patients; (d) the lack of rating scales measuring specifically vortioxetine DS; and (e) the naturalistic open-label design of the study without placebo control and randomization.

## 4. Materials and Methods

**Figure 1:**
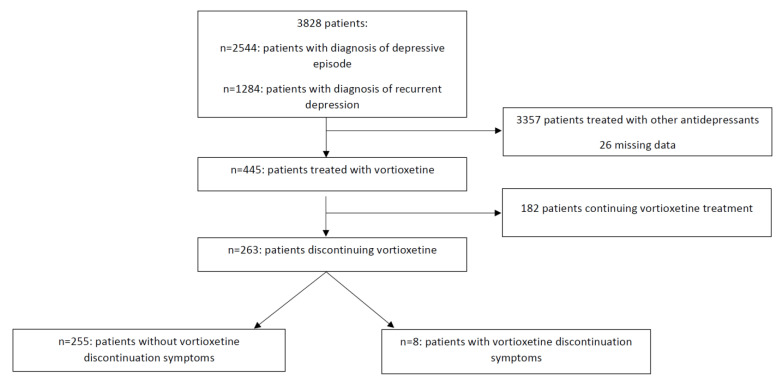
Flow chart of retrospective chart review.

A retrospective chart review was performed to evaluate the prevalence and clinical characteristics of vortioxetine treatment discontinuation symptoms. Analysis was performed by all of the authors. The dataset included paper and electronic medical records of all of the patients diagnosed with a depressive episode or recurrent depressive disorder according to ICD-10 treated in the Department of Adult Psychiatry of University Hospital in Cracow between 2014–2020. Patients’ data were selected for further analysis if they met the following inclusion criteria: (1) age over 18, (2) received treatment with vortioxetine in monotherapy or in combination with another psychotropic drug, and (3) discontinued vortioxetine (for any reason). Figure 1 presents a flow chart of retrospective chart review. In summary, documentation of 3828 patients (2544 with the diagnosis of depressive episode and 1284 with the diagnosis of recurrent depressive disorder) were evaluated. A total of 445 patients from this group received vortioxetine treatment, of which 263 stopped the medication.

Data were extracted from medical records of patients discontinuing vortioxetine with the use of chart instrument in the form of electronic table file that encoded the following variables: age, sex, depression diagnosis, somatic comorbidities, psychiatric comorbidities, presence of comorbid anxiety disorders (general anxiety disorder, panic disorder, social anxiety disorder, somatoform disorder, agoraphobia, and/or other unspecified anxiety disorders according to ICD-10), type of therapy (monotherapy/polytherapy), days of pharmacotherapy prior to discontinuation, method of drug withdrawal (sudden or gradual), the way of deciding on discontinuation (with/without medical consultation), reason for discontinuation (due to side effects, lack of effectiveness, symptomatic improvement/remission, accidental discontinuation, others), switch to different antidepressant medication (with SSRI/SNRI mechanism of action, other than SSRI/SNRI mechanism of action or cessation of antidepression pharmacotherapy), and occurrence of withdrawal symptoms. Ambiguous cases were resolved by the consensus of all of the authors of this article.

Furthermore, we examined the clinical characteristics of patients with vortioxetine DS, including their depressive symptoms before initiation of treatment and the course and symptoms of withdrawal syndrome. Vortioxetine DS have been evaluated with the use of the Polish 43-item, clinician-rated checklist version of the Discontinuation-Emergent Signs and Symptoms (DESS) inventory [28].

### Data Analysis

Relationship between sex, type of pharmacotherapy (monotherapy/polytherapy), way of deciding on discontinuation of treatment (with/without medical consultation), method of discontinuation (sudden or gradual), somatic comorbidities, number of psychiatric comorbidities, presence of comorbid anxiety disorders, and occurrence of withdrawal symptoms was evaluated with the use of Fisher’s exact tests.

Associations between age, duration of vortioxetine pharmacotherapy, dose, and occurrence of withdrawal symptoms were evaluated with the use of U-Mann–Whitney tests. The estimated effect size was calculated by the formula from Rosenthal (1991) [29]. The relationship between the reason for discontinuing vortioxetine, switch to different antidepressant medication, and occurrence of withdrawal symptoms were evaluated with the use of Fisher–Freeman–Halton’s test. False discovery rate (FDR) correction with Benjamin–Hochberg procedure was used in order to adjust the *p*-value for multiple comparisons.

## 5. Conclusions

The good tolerance and efficacy of vortioxetine in the treatment of depressive disorders are well documented. However, just like during withdrawal of other antidepressants, there is a possibility of DS emergence upon vortioxetine cessation (even when the lowest therapeutic dose is administered, and the drug is gradually tapered off). Taking into consideration that vortioxetine is a relatively new antidepressant in the pharmaceutical market, further studies of discontinuation syndrome developing after stopping this medication are necessary.

## Figures and Tables

**Table 1:** Clinical characteristics of groups with and without discontinuation symptoms after vortioxetine therapy.

KERRYPNX	Patients without Vortioxetine Discontinuation Symptoms (*n* = 255)	Patients with Vortioxetine Discontinuation Symptoms (*n* = 8)	*p*
Age (median number of years, (25th percentile–75th percentile))	39 (30–51)	31 (27–52.8)	*p*_Mann–Whitney_ = 0.326
Sex (women/men)	143/112	5/3	*p*_Fisher’s exact test_ > 0.999
Therapy (% of patients with monotherapy)	23.5%	62.5%	*p*_Fisher’s exact test_ = 0.024
Vortioxetine dose (mean mg, (SD))	10.58 (2.38)	11.25 (2.32)	*p*_Mann–Whitney_ = 0.057
Somatic comorbidities (%)	20%	37.5%	*p*_Fisher’s exact test_ = 0.212
Number of psychiatric comorbidities			*p*_Fisher’s exact test_ = 0.947
0	36.9%	50%	
1	36.1%	37.5%	
2	19.6%	12.5%	
3	5.9%	0%	
4	1.6%	0%	
Comorbid anxiety disorders (% of patients)	42%	50%	*P*_Fisher’s exact test_ = 0.725
Days of pharmacotherapy prior to discontinuation (median number of days, (25th percentile–75th percentile))	74 (38.8–170.3)	272 (71–375)	*p*_Mann–Whitney_ = 0.032
Discontinuing: gradual or sudden (*n*, (%) of sudden)	208 (81.6%)	5 (62.5%)	*p*_Fisher’s exact test_ = 0.179
Discontinuing: with or without prior medical advice (*n*, (%) of without)	47 (18.4%)	5 (62. 5%)	*p*_Fisher’s exact test_ = 0.009 *
Reason for discontinuation (*n*, (%))			*p*_FFH_ < 0.001 *
Side effects	40 (17.3%)	1 (14.3%)	
Lack of effectiveness	135 (58.4%)	0 (0%)	
Symptomatic improvement/remission	32 (13.9%)	2 (28.5%)	
Accidental discontinuation	2 (0.9%)	4 (57.1%)	
Others	22 (9.5%)	0 (0%)	
Switch to different antidepressant medication (*n*, (%))			*p*_Fisher’s exact test_ = 0.003 *
SSRI	134 (52.5%)	0 (0%)	
Other	37 (14.5%)	2 (25%)	
No switch	69 (27%)	6 (75%)	
Median DESS score (range)		4 (4–6)	
Median number of days between vortioxetine withdrawal and emergence of DS (*n*, (25th percentile–75th percentile))	-	3 (1.25–4.75)	

FFH, Fisher–Freeman–Halton’s Test; DESS, Discontinuation-Emergent Signs and Symptoms inventory. DS, discontinuation symptoms. *, comparisons that remained statistically significant after implementing false discovery rate (FDR) *p*-value correction with Benjamin–Hochberg procedure for multiple comparisons.

**Table 2:** Discontinuation symptoms after discontinuation of vortioxetine therapy.

DS Symptom	No. of Patients (%)
DESS symptoms	
Lability	8 (100%)
Irritability	6 (75%)
Sudden worsening of mood	6 (75%)
Nervousness	3 (38%)
Agitation	3 (38%)
Tearfulness	2 (25%)
Headache	2 (25%)
Concentration deficits	1 (13%)
Stomach cramps	1 (13%)
Sleep disorders	1 (13%)
Tremor	1 (13%)
Dizziness	1 (13%)
Hypersensitivity to stimuli	1 (13%)
Symptoms not represented in DESS	
Anergy	2 (25%)
Apathy	1 (13%)
Amotivation	1 (13%)
Physical weakness	1 (13%)

DESS, Discontinuation-Emergent Signs and Symptoms inventory; DS, discontinuation symptoms.

## Data Availability

The data presented in this study are available on request from the corresponding author.

## References

[1] Grohol J. Top 25 Psychiatric Medication Prescriptions for 2013. https://psychcentral.com/lib/top-25-psychiatric-medication-prescriptions-for-2013/.

[2] Mann A.M., Macpherson A.S. (1959). Clinical experience with imipramine (G22355) in the treatment of depression. Can. Psychiatr. Assoc. J..

[3] Keks N., Hope J., Keogh S. (2016). Switching and stopping antidepressants. Aust. Prescr..

[4] Heitzman J., Solak M. (2009). Zespół objawów odstawiennych po lekach antydepresyjnych w badaniach naukowych-Implikacje dla lekarzy i pacjentów. Psychiatr. Pol..

[5] Warner C.H., Bobo W., Warner C., Reid S., Rachal J. (2006). Antidepressant discontinuation syndrome. Am. Fam. Physician.

[6] Schweitzer I., Maguire K. (2001). Stopping antidepressants. Aust. Prescr..

[7] Haddad P.M., Anderson I.M. (2007). Recognising and managing antidepressant discontinuation symptoms. Adv. Psychiatr. Treat..

[8] Ivd W., Ashby D., Brook R., Mga C., Chick J., Drummond C., Ebmeier K.P., Gunnell D., Hawking H., Mukaetova-Ladinska E. (2005). Report of the CSM Expert Working Group on the Safety of Selective Serotonin Reuptake Inhibitor Antidepressants.

[9] Carvalho A.F., Sharma M.S., Brunoni A.R., Vieta E., Fava G.A. (2016). The Safety, Tolerability and Risks Associated with the Use of Newer Generation Antidepressant Drugs: A Critical Review of the Literature. Psychother. Psychosom..

[10] Fava G.A., Gatti A., Belaise C., Guidi J., Offidani E. (2015). Withdrawal symptoms after selective serotonin reuptake inhibitor discontinuation: A systematic review. Psychother. Psychosom..

[11] Campagne D.M. (2005). Venlafaxine and serious withdrawal symptoms: Warning to drivers. Med. Gen. Med..

[12] Perahia D.G., Kajdasz D.K., Desaiah D., Haddad P.M. (2005). Symptoms following abrupt discontinuation of duloxetine treatment in patients with major depressive disorder. J. Affect. Disord..

[13] Curtin F., Berney P., Kaufmann C. (2002). Moclobemide discontinuation syndrome predominantly presenting with influenza-like symptoms. J. Psychopharmacol..

[14] https://www.accessdata.fda.gov/drugsatfda_docs/label/2016/020415s030lbl.pdf.

[15] Verma J.K., Mohapatra S. (2015). Mirtazapine withdrawal-induced mania. J. Pharmacol. Pharmacother..

[16] Berigan T.R. (2001). Mirtazapine-Associated Withdrawal Symptoms: A Case Report. Prim. Care Companion J. Clin. Psychiatry.

[17] Kuniyoshi M., Arikawa K., Miura C., Inanaga K. (1989). Panic Anxiety after Abrupt Discontinuation of Mianserin. Psychiatry Clin. Neurosci..

[18] Plesničar B.K. (2014). Efficacy and tolerability of agomelatine in the treatment of depression. Patient Prefer. Adherence.

[19] Stoukides J.A., Stoukides C.A. (1991). Extrapyramidal symptoms upon discontinuation of fluoxetine. Am. J. Psychiatry.

[20] Narayan V., Haddad P.M. (2011). Antidepressant discontinuation manic states: A critical review of the literature and suggested diagnostic criteria. J. Psychopharmacol..

[21] Blum D., Maldonado J., Meyer E., Lansberg M. (2008). Delirium following abrupt discontinuation of fluoxetine. Clin. Neurol. Neurosurg..

[22] Van Noorden M.S., Vergouwen A.C.M., Koerselman G.F. (2002). Delirium bij afbouw van venlafaxine. Ned. Tijdschr. Geneeskd..

[23] Wang J., Greenberg H. (2013). Status cataplecticus precipitated by abrupt withdrawal of venlafaxine. J. Clin. Sleep Med..

[24] Sabljić V., Ružić K., Rakun R. (2011). Venlafaxine withdrawal syndrome. Psychiatr. Danub..

[25] Haddad P., Devarajan S., Dursun S. (2001). Antidepressant discontinuation (withdrawal) symptoms presenting as “stroke”. J. Psychopharmacol..

[26] Jacobsen P.L., Mahableshwarkar A.R., Serenko M., Chan S., Trivedi M.H. (2015). A randomized, double-blind, placebo-controlled study of the efficacy and safety of vortioxetine 10 mg and 20 mg in adults with major depressive disorder. J. Clin. Psychiatry.

[27] Baldwin D.S., Chrones L., Florea I., Nielsen R., Nomikos G.G., Palo W., Reines E. (2016). The safety and tolerability of vortioxetine: Analysis of data from randomized placebo-controlled trials and open-label extension studies. J. Psychopharmacol..

[28] Cubała W., Landowski J., Springer J. (2013). Discontinuation-emergent signs and symptoms inventory-Polish translation of the discontinuation signs and symptoms checklist. Psychiatr. Pol..

[29] Rosenthal R. (2011). Meta-Analytic Procedures for Social Research.

[30] Zhang J., Mathis M.V., Sellers J.W., Kordzakhia G., Jackson A.J., Dow A., Yang P., Fossom L., Zhu H., Patel H. (2015). The US Food and Drug Administration’s perspective on the new antidepressant vortioxetine. J. Clin. Psychiatry.

[31] Rosenbaum J.F., Fava M., Hoog S.L., Ascroft R.C., Krebs W.B. (1998). Selective serotonin reuptake inhibitor discontinuation syndrome: A randomized clinical trial. Biol. Psychiatry.

[32] Sowa-Kućma M., Pańczyszyn-Trzewik P., Misztak P., Jaeschke R.R., Sendek K., Styczeń K., Datka W., Koperny M. (2017). Vortioxetine: A review of the pharmacology and clinical profile of the novel antidepressant. Pharmacol. Rep..

